# Evaluating Conservation Corridor Success for Rare and Common Dragonflies Using Zeta Diversity

**DOI:** 10.1002/ece3.73251

**Published:** 2026-03-29

**Authors:** Gabriella J. Kietzka, James S. Pryke, Rene Gaigher, Michael J. Samways, Cang Hui

**Affiliations:** ^1^ Mathematical Biosciences Hub, Department of Mathematical Sciences Stellenbosch University Stellenbosch South Africa; ^2^ Department of Conservation Ecology and Entomology Stellenbosch University Stellenbosch South Africa; ^3^ Biodiversity Informatics Unit African Institute for Mathematical Sciences Cape Town South Africa; ^4^ Centre for Invasion Biology National Institute for Theoretical and Computational Sciences Stellenbosch South Africa

**Keywords:** biodiversity, grasslands, multisite turnover, odonata, plantation forestry, species distribution

## Abstract

Conservation corridors connect natural areas, aiming to mitigate the effects of land transformation. However, their influence on biodiversity, particularly species turnover, remains poorly understood. This study evaluates the impact of conservation corridors on riverine ecosystems and their associated dragonfly assemblages. We assessed species richness and applied the zeta diversity framework to evaluate species turnover across multiple sites, thereby providing insights into how these corridors influence dragonfly community composition relative to natural areas. The research was conducted in the KwaZulu‐Natal Midlands of South Africa, covering 104 freshwater sites within natural grasslands and timber plantation corridors. At each site, a 100 m transect adjacent to a river was sampled twice, focusing on recording adult male dragonflies and six environmental variables. Drivers of species richness were analysed using generalised additive models and generalised linear models. Multi‐site generalised dissimilarity models were run to examine changes in zeta diversity along environmental gradients and to partition the contributions of different factors to compositional turnover. A total of 37 species were recorded, with one species exclusive to natural areas and four unique to corridors. Dragonfly assemblages were influenced more by stochastic processes than by environmental gradients. Although factors such as site distance, differences in water temperature, dissolved oxygen, shade and rock cover affected turnover, they explained little variation in both rare and common species. Species richness was higher in corridors and consistently declined with increasing shade cover. Neither the presence of corridors nor invasive alien vegetation influenced species turnover, indicating that corridors function similarly to natural habitats. This study demonstrates the crucial role of conservation corridors in preserving dragonfly diversity in altered landscapes. Our findings support continued investment in corridor implementation and management for biodiversity conservation and demonstrate the utility of the zeta diversity framework for understanding species turnover dynamics.

## Graphical abstract

This study applied the zeta diversity framework to evaluate how conservation corridors in South African timber plantations impact dragonfly assemblages, finding that corridors function similarly to natural habitats. The research findings showed that dragonfly species richness was higher in corridors than in natural areas, consistently declining with increased shade cover, and with species turnover being influenced more by stochastic processes than environmental factors. These findings support continued investment in conservation corridors as effective tools for preserving biodiversity in transformed landscapes, while demonstrating the value of zeta diversity analysis for understanding species turnover patterns across multiple sites.

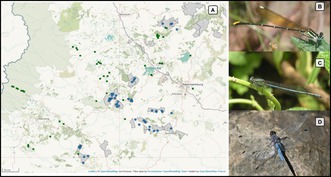

## 1 Introduction

For successful biodiversity preservation, it is crucial to assess conservation actions aimed at offsetting the negative impacts associated with land‐use change (Baldwin et al. 2018). One example is conservation corridors (hereafter referred to as corridors), which are established to mitigate the effects resulting from land transformation, such as habitat loss and fragmentation that lead to declines in biodiversity (Beier 2012). In this context, a corridor typically refers to a linear strip of natural grassland between plantation stands, acting as a link connecting two or more larger, untransformed natural areas. Well‐managed corridors are characterised by interventions that successfully preserve a vegetation structure akin to that of natural grasslands. These corridors are heterogeneous, sufficiently expansive to minimise the impacts of adjacent plantations and with minimal invasion by alien plant species. Corridors thereby facilitate the survival and dispersal of organisms in transformed landscapes (Boitani et al. 2007; Samways et al. 2025; Travers et al. 2021).

Corridors have been established in some South African plantations to reduce the detrimental effects caused by converting grasslands into monospecific stands of alien trees (Samways et al. 2010). The use of corridors for dispersal and/or as suitable habitat varies across taxa and depends on attributes related to species mobility, ecology, habitat sensitivity and behaviour (Samways et al. 2010). Additionally, maintaining heterogeneity in an ecosystem is crucial, as species have diverse requirements and respond differently to the structural and functional components of corridors and landscapes (Pryke and Samways 2015). The goal here is to establish and maintain corridors that resemble natural areas in terms of habitat quality and heterogeneity to support the full suite of resident species.

We must identify the requisites for supporting the complete range of species during the design and management of corridors, encompassing the needs of both rare, narrow‐ranged species and common, widespread species. Rare and common species fulfill distinct roles in ecosystems, with rare species contributing significantly to functional diversity, enhancing ecosystem resilience, whereas common species often drive ecosystem processes due to their wide distributions and high abundances (Mouillot et al. 2013; Zhang et al. 2022). Both are needed for maintaining ecosystem stability and health, underscoring the importance of minimising turnover from landscape transformation for all species, both rare and common (Chapman et al. 2018; Latombe et al. 2017).

Species turnover has been used to assess corridor success for conservation, typically through methods related to beta diversity, which involve comparisons of species lists between pairs of sites (Pardini et al. 2005; Samways et al. 2010; Tockner and Ward 1999). However, these methods may not represent the full complement of biodiversity partitions across multiple sites (McGeoch et al. 2019). Between pairs of sites, compositional similarity is primarily driven by the gain and loss of rare species (such as singletons and doubletons). Yet, these rare species are precluded from contributing to higher order similarity especially when three or more sites are considered. To overcome such biodiversity information leakage in pairwise turnover, the concept of zeta diversity was proposed (Hui and McGeoch 2014).

Zeta diversity quantifies assemblage turnover by considering how the number of shared taxa varies across a given number of sites, thereby distinguishing the roles of rare and common taxa in compositional diversity. Specifically, zeta decline describes how the number of species shared by multiple assemblages decreases with an increasing number of sites (Latombe et al. 2017; Latombe, McGeoch, et al. 2018; McGeoch et al. 2017). A steep zeta decline signifies turnover driven by rare species, while a shallow decline indicates turnover driven by more common species (McGeoch et al. 2019). The shape of the zeta decline, which is either power‐law or exponential, is also indicative of community assembly processes (Hui and McGeoch 2014).

The value of the zeta diversity framework extends beyond inferring assemblage composition and identifying drivers of species turnover (Hui and McGeoch 2014; Kunin et al. 2018). When used in conservation assessment and management, this cross‐site comparison of species composition can provide evidence on how insect biodiversity responds to environmental stressors (Benites et al. 2025; Parra‐Sanchez et al. 2025) and support the evaluation of conservation actions, as demonstrated in various studies across different taxa. To list a few, zeta diversity has been used to identify the factors needed to optimise networks of nature reserves for plants in the Czech Republic (Latombe, Richardson, et al. 2018) and for mammals in China (Ke et al. 2023; Wen et al. 2022). It has also been used to assess the efficiency of marine protected areas for conserving fish diversity in Australia (Pettersen et al. 2022).

Insects are recognised as promising bioindicators due to their substantial contributions to global biomass, species richness and ecological function, making them suitable candidates for assessing the effectiveness of conservation strategies (Chowdhury et al. 2023; McGeoch 2007; Relyea et al. 2000). Further to this, many are well‐studied, have diverse life histories and are highly responsive to environmental change (McGeoch 2007). By applying the zeta diversity framework to bioindicator taxa, we evaluate the success of conservation strategies in maintaining the integrity of species assemblages, not only within the focal group but potentially across entire ecosystems.

The insect order Odonata comprises dragonflies and damselflies (hereafter collectively referred to as dragonflies), which are extensively used as bioindicators and are model organisms for representing biodiversity and ecosystem conditions, as well as being umbrella species (Bried et al. 2007; Bulánková 1997; Chovanec and Raab 1997; Clark and Samways 1996; Kietzka et al. 2019; Rocha‐Ortega et al. 2019). Here, we apply the zeta diversity framework to assess the effectiveness of corridors in conserving dragonfly diversity by reducing turnover of rare and common species in an essentially transformed forestry landscape. Specifically, we aim to determine whether rivers within corridors show patterns of dragonfly diversity and environmental drivers equivalent to those of nearby natural, untransformed areas, for both rare and common species. We expect that rivers within corridors will reflect similar patterns and drivers of dragonfly diversity as natural, untransformed grassland areas, but that rare species will be more sensitive to anthropogenic impacts.

## 2 Methods

### 2.1 Site Details and Data Collection

**FIGURE 1:**
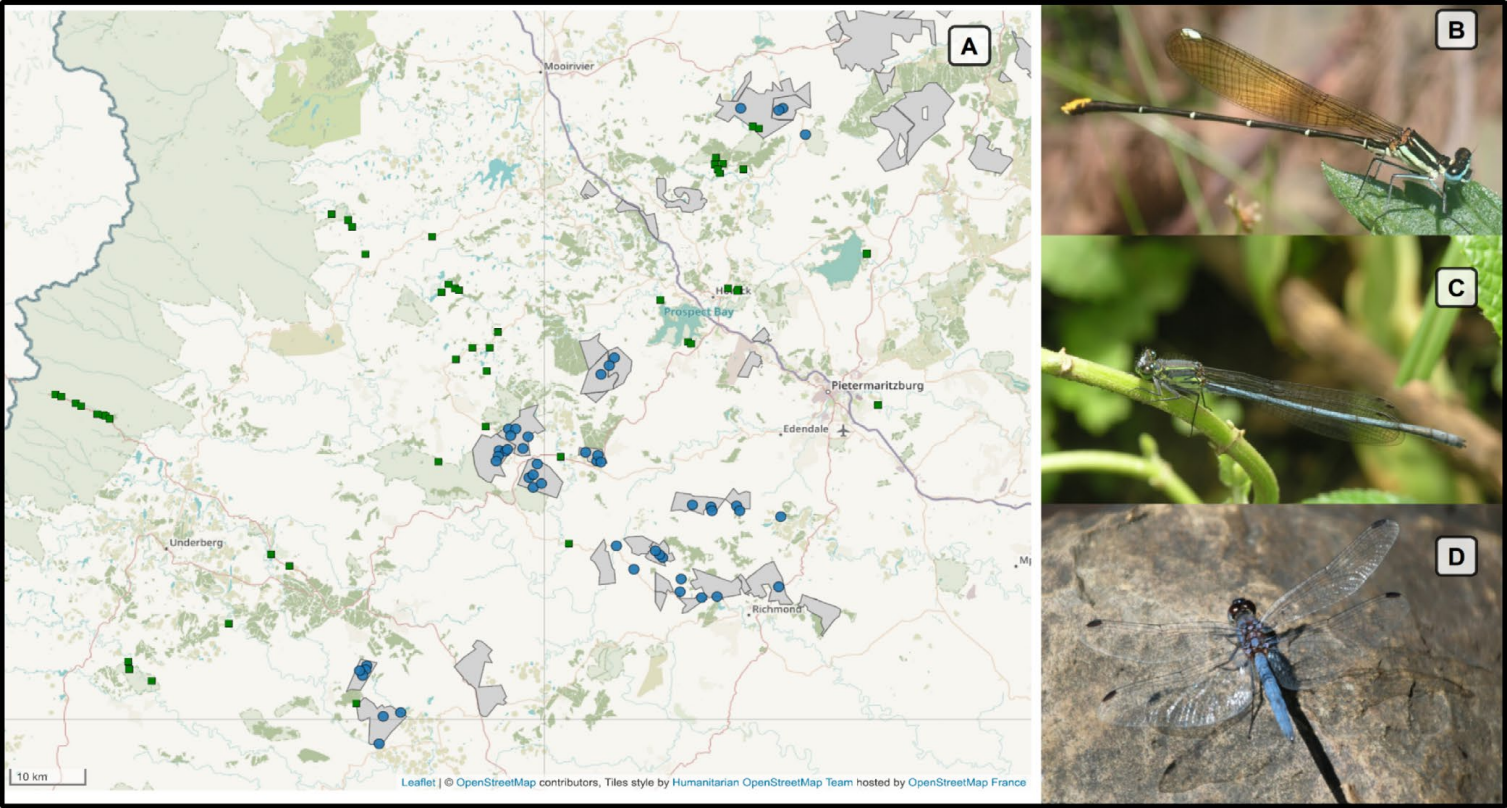
(A) Sampled sites in the KwaZulu‐Natal Province of South Africa. Green‐filled squares represent natural sites and blue‐filled circles represent corridor sites in plantations (plantations are outlined and shaded in grey). Plantation boundary data were obtained from the Mondi Group. The most frequently observed Odonata species included: (B) *Allocnemis leucosticta*, (C) *Pseudagrion spernatum* and (D) *Trithemis furva*. See Data S8 for site photos.

Research was conducted across diverse commercial timber plantations in the KwaZulu‐Natal Midlands, South Africa (Figure 1). The region has a temperate climate with predominantly summer rainfall patterns, from September to April. The area experiences significant temperature variations, ranging from 2°C to 38.8°C, with a mean annual temperature of approximately 15°C and an average annual precipitation of around 900 mm. The diverse topography encompasses steep mountain valleys and rolling grassland hills, with elevations ranging from 800 to 1800 m above sea level (Rutherford et al. 2006). The landscape is characterised by hills, rocky outcrops and clay soils (Pryke and Samways 2015). The plantations primarily consist of *Eucalyptus* and *Pinus* species and the natural vegetation comprises two prominent types: the endangered Midlands Mistbelt Grassland and the Drakensberg Foothill Moist Grassland (Rutherford et al. 2006). The selected sites aimed to capture a wide range of variations in corridor characteristics and natural reference sites were streams or rivers (referred to collectively as rivers) located within protected areas or those surrounded by extensive natural vegetation. River sites were selected according to two land‐use categories (natural grasslands in protected areas and corridors in commercial plantations). Each site comprised a 100 m transect along the edge of a river, randomly selected to represent the range of environmental conditions, rather than for comparability, to reflect the true heterogeneity of the landscape. As expected, site distribution was uneven. For example, a cluster of natural sites is evident along the Sani Pass Road at high elevations. To address such differences, modelling approaches were used that incorporated both geographic distance and environmental gradients. A total of 104 freshwater river sites were selected (Figure 1). Of these sites, 55 were in natural, untransformed grasslands and 49 were in corridors. For detailed data collection methods, see Kietzka et al. (2021a).

From January to March 2018, each site was sampled twice (2 h in total) by two observers. Our study was conducted during a single summer season, which captures the peak activity period for most adult dragonfly species in the region but does not account for interannual variation. Adult male dragonfly species and abundances were recorded from 9 h00–15 h00, when conditions were warm and cloud cover was less than 30%. We use male individuals because they are territorial and always occur at their waterside territories; and unlike many females (and young adults), they are vibrantly coloured, and their conspicuous bodies make it possible for species‐level identification (Samways and Simaika 2016). Individuals that were difficult to identify were captured with handheld nets and identified according to Samways and Simaika (2016); voucher specimens are retained at the Entomological Museum at Stellenbosch University, South Africa.

The selected environmental variables included: shade cover, water temperature, water dissolved oxygen, rock cover, percentage of invasive alien vegetation and land use (Table 1). The selection of these environmental variables was based on previous research in the same region using 18 variables (Kietzka et al. 2021a), which included some of the same sites. These selected variables reflect both riverine conditions and corridor characteristics that are known for their known influence on dragonfly assemblages as well as their relevance to corridor design and management. For instance, shade cover serves as a proxy for corridor width and vegetation structure and influences habitat selection and adult abundance (French and McCauley 2018; Remsburg and Turner 2009). Water temperature is a key determinant of species richness and activity patterns (Kietzka et al. 2017; McCauley et al. 2018). Dissolved oxygen levels affect larval survival and community composition (Kietzka et al. 2017; Lee and Matthews 2024; Mendes et al. 2018) and substrate type, such as rock cover, provides essential larval habitat and is important for thermoregulation (Kietzka et al. 2021b; Lubertazzi and Ginsberg 2009; Worthen and Horacek 2015).

**TABLE 1:** Details of the selected variables and recording methods used. *A* = all sites together, *N* = natural sites, *C* = corridor sites.

Variable	Method	Mean (SD)
Shade cover (%)	At midday, estimated percentage cover by two observers and averaged	*A*: 29 (31), *N*: 22 (29), *C*: 36 (32)
Water temperature (°C)	Combo Meter (Model: HI98129; Make: HANNA Instruments)	*A*: 23 (3), *N*: 23(3), *C*: 22 (3)
Dissolved oxygen (%)	Handheld dissolved oxygen meter (Model: HI9146; Make: HANNA Instruments)	*A*: 78 (13), *N*: 73 (7), *C*: 83 (16)
Rock cover (%)	Estimated percentage cover of any visible rocks (Peninsula Formation Sandstone) at river edges by two observers and averaged	*A*: 40 (36), *N*: 41 (37), *C*: 39 (35)
Alien vegetation (%)	Estimated percentage based on the most common species in the area, which included (in order of prevalence across sites): American bramble ( *Rubus cuneifolius* ), black wattle ( *Acacia mearnsii* ), bugweed ( *Solanum mauritianum* ) and scotch thistle ( *Cirsium vulgare* ).	*A*: 33 (35), *N*: 22 (30), *C*: 45 (36)
Land use (category)	Whether a site was situated in a natural, undisturbed area or a corridor in a plantation	

Invasive alien vegetation can create unnatural shading, degrade water quality and flow dynamics and reduce microhabitat availability, negatively impacting dragonfly assemblages (Kietzka et al. 2021a, 2021b; Samways and Sharratt 2010), Invasive vegetation cover also indicates management effectiveness. We calculated invasive alien vegetation cover based on the most common species in the area, which included: American bramble ( *Rubus cuneifolius* ), black wattle ( *Acacia mearnsii* ), bugweed ( *Solanum mauritianum* ) and scotch thistle ( *Cirsium vulgare* ). Land use types (natural areas vs. corridors) were also included, as they strongly influence both adult and larval dragonfly communities by affecting connectivity, water quality and habitat complexity (Goertzen and Suhling 2019; Luke et al. 2017). All selected variables were measured twice per site. For additional information on how these variables were measured, see Table 1.

### 2.2 Multisite Zeta Diversity

Broadscale assembly processes of dragonflies can be determined by examining changes to the zeta diversity values with increasing order (number of sites considered). It is termed zeta decline, which was calculated using the zeta decline function in the *zetadiv* package v1.2.1 (Latombe, McGeoch, et al. 2018) for the first five orders of zeta (ζ_1_–ζ_5_). Order of zeta, indicated as a subscript, refers to the number of sites considered. Zeta decline can provide a broad indication of whether compositional turnover is primarily driven by deterministic or stochastic processes. Analyses were run for dragonfly assemblages of natural and corridor sites combined and separately.

To understand the determinants of within‐site dragonfly diversity, we used the first order of zeta (ζ_1_) as the response variable, which represents the number of species at a site, or species richness. Generalised additive models (GAMs) with restricted maximum likelihood (REML) estimation from the *mgcv* package (Wood and Wood 2015) and generalised linear models (GLMs) were run to determine which variables drive changes in dragonfly species richness. GAMs account for non‐linearities and non‐monotonicity, whereas GLMs indicate whether the overall effect of a predictor is positive or negative. Both methods were run for all sites combined and included land use (natural or corridor) as one of the six explanatory variables (Table 1). For GAMs, the predictors were checked for concurvity and those with a ’worst’ value > 0.75 were removed in a sequential fashion. Species richness is considered count data and thus we chose the log‐link function and the quasi‐Poisson family for both GAMs and GLMs.

Multi‐site generalised dissimilarity models (MS‐GDMs) were run to determine how zeta diversity (turnover) changes with environmental gradients represented by five natural and anthropogenic predictors (shade cover, water temperature, water dissolved oxygen, rock cover and percentage of invasive alien vegetation), plus land use (natural or corridor) and geographic distance between sites. An MS‐GDM implements generalised dissimilarity modelling of zeta diversity to determine drivers of compositional turnover for different orders, with higher orders reflecting turnover from increasingly more widespread species. To assess true turnover arising from species gains and losses, rather than artefacts of nested species lists among sites, we used Simpson‐equivalent zeta diversity (a normalised metric reflecting true turnover, bounded between 0 and 1; Latombe, McGeoch, et al. 2018). This metric was implemented in the MS‐GDM using 3000 random combinations of sites for zeta orders two and five (ζ_2_ and ζ_5_). We focused on orders two and five to represent opposite ends of the commonness spectrum. Zeta order two (ζ₂) reflects turnover dominated by rare species (equivalent to traditional beta diversity), while order five (ζ₅) represents turnover of more widespread species that occur across multiple sites. This approach thus allows us to distinguish between factors affecting rare versus common species. To ease comparisons with the models for species richness, we also used GLM and GAM methods in the MS‐GDM to test the response of dragonfly species turnover to predictor gradients.

Furthermore, assuming distance decay of compositional and environmental similarity, we also conducted I‐spline regression in the MS‐GDM to account for overall and local responses of compositional turnover to gradients of the selected variables (Ferrier et al. 2007; Latombe, McGeoch, et al. 2018; McGeoch et al. 2017). The I‐spline regression technique, implemented with three knots, allows us to identify which portions of environmental gradients (low, medium or high values) most strongly influence species turnover. For example, this approach can reveal whether small differences in water temperature at the cooler end of the gradient have stronger effects on community composition than similar differences at the warmer end. For these MS‐GDM analyses, logit link function and quasi‐binomial family were utilised due to the bounded distribution of the Simpson‐equivalent zeta diversity. All analyses were conducted in R version 4.3.3 (R Core Team 2024).

## 3 Results

### 3.1 Zeta Diversity Decline

Zeta diversity declined to below one after only order two. This means that, on average, less than one species was shared among two or more sites (Table 2; Data S1). However, corridor sites showed slightly higher species sharing compared to overall or natural sites (Table 2). This indicates that although corridors may support natural processes similar to those in relatively intact environments, including facilitating the movement of dragonflies between sites to acquire resources, they may be slightly more homogeneous in species composition than natural habitats.

**TABLE 2:** Mean and standard deviation of zeta diversity for orders from one to five for dragonflies in natural and corridor sites overall and separately. SD = standard deviation.

Zeta order	Mean zeta (SD)		
	Overall	Natural	Corridors
1	4.03 (2.24)	3.89 (2.13)	4.41 (2.72)
2	0.80 (0.92)	0.68 (0.89)	1.03 (1.08)
3	0.21 (0.46)	0.15 (0.40)	0.32 (0.59)
4	0.06 (0.25)	0.04 (0.20)	0.11 (0.34)
5	0.02 (0.14)	0.01 (0.10)	0.04 (0.20)

Zeta diversity decline of dragonfly species conformed best to an exponential form for the first five orders of zeta, rather than a power‐law form (Table 3). An exponential decline in zeta diversity suggests that each species has an equal probability of occurring at a site. This shows that the presence of each species reflects more stochastic processes (chance), rather than deterministic mechanisms (environmental filtering due to niche differentiation). It may also indicate that all detected species have narrow ranges, such that their occurrence at a site does not fully capture shifts in habitat preference across the surveyed environmental gradients. Alternatively, the 2 h sampling duration per site may have been insufficient to reliably detect species‐environment associations.

**TABLE 3:** Corrected goodness‐of‐fit and Akaike information criterion (AIC) of model fitting for exponential versus power‐law regressions of zeta diversity decline for dragonflies in natural and corridor sites overall and separately.

Metric	Land use		
	Overall	Natural	Corridors
Exponential adj R^2^	0.994***	0.996***	0.994***
AIC	−9.10	−9.90	−9.76
Power‐law adj R^2^	0.963**	0.959**	0.965**
AIC	0.32	1.89	−1.36

*Note:* Significant *p*‐value levels: * *p* < 0.05, ** *p* < 0.01, *** *p* < 0.001 (Cowles and Davis (1982)).

### 3.2 Species Richness

Of the 37 dragonfly species recorded, 18 Anisoptera and 15 Zygoptera species occurred in natural sites, while 20 Anisoptera and 16 Zygoptera species occurred in corridor sites (Data S2). One species, *Pseudagrion hageni*, was unique to natural sites, and four species were exclusive to corridors, which included *Africallagma sapphirinum*, *Tramea basilaris*, *Crocothemis erythraea* and notably a rare and highly threatened species, *Chlorolestes apricans* (Data S2).

**FIGURE 2:**
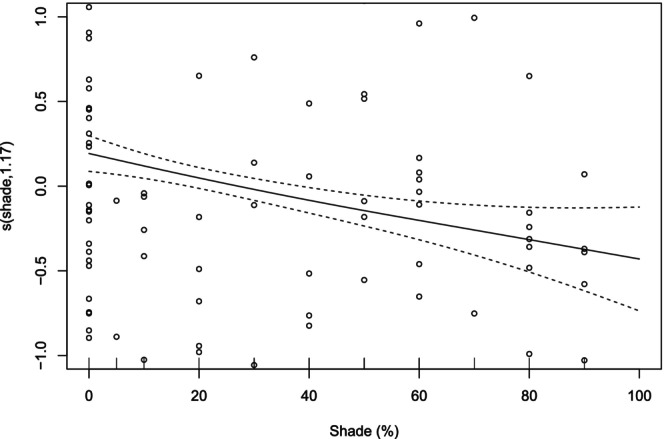
Response curve from the generalised additive model (GAM) for shade as a significant predictor of species richness.

**FIGURE 3:**
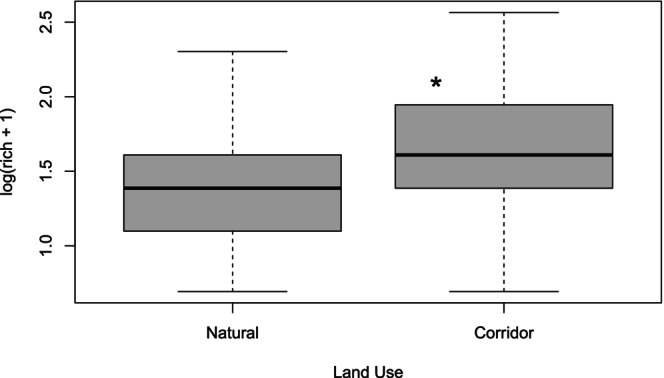
Boxplot of significantly different species richness (log of richness plus one) between land use types (Natural and Corridor). Significantly higher richness represented by * (*p* < 0.05).

In the case of natural sites, the three most widespread species included *Allocnemis leucosticta*, *Trithemis furva* and *Pseudagrion spernatum*, although their occupancies (proportion of sites occupied) were low, 0.36, 0.35 and 0.27, respectively. For corridor sites, *P. spernatum* and *A. leucosticta* were also in the top three most widespread species, in addition to *Pseudagrion caffrum* (Data S2), also with low occupancies (0.47, 0.43 and 0.37, respectively). For predictors of species richness, the deviance explained by the GAM was 24.6%, with the results also corroborated by those from the GLM (18.5% variance explained). Both models showed that shade cover and land use were the significant variables that explained the variation of dragonfly species richness. Increasing shade cover reduced species richness in a weakly non‐linear form, while species richness was significantly higher in corridors than in natural sites (Figures 2 and 3; Data S3 and S4).

### 3.3 Compositional Turnover

**FIGURE 4:**
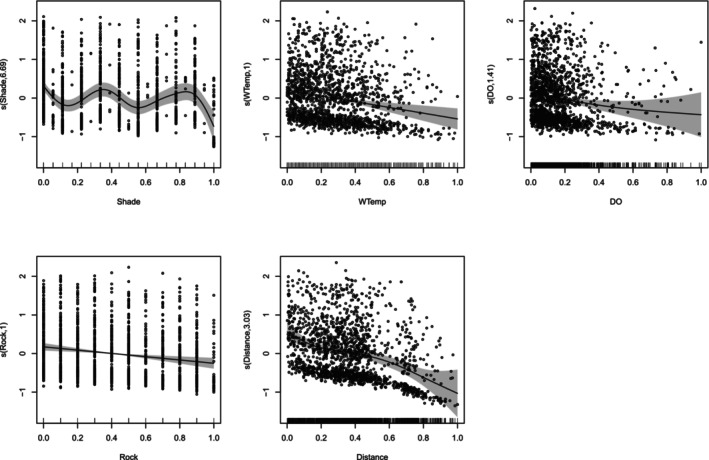
Significant predictors of species turnover identified using the GAM method of the MS‐GDM, with compositional similarity measured by normalised zeta diversity (Simpson‐equivalent) for rare species (ζ_2_). Labels on the y‐axis correspond to the component of the smoothing function (*s*) in the GAM, with the estimated degrees of nonlinearity. The ticks on the x‐axis represent 3000 site pairs. Grey shading depicts the confidence intervals at the 95% level.

**FIGURE 5:**
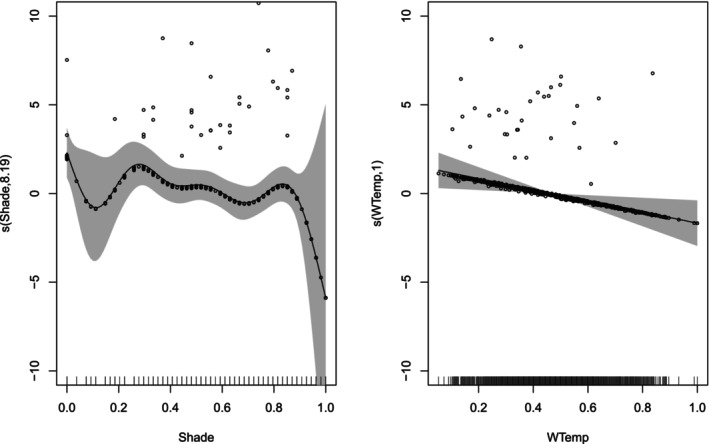
Significant predictors of species turnover identified using the GAM method of the MS‐GDM, with compositional similarity measured by normalised zeta diversity (Simpson‐equivalent) for common species (ζ_5_). Labels on the y‐axis correspond to components of the smoothing function (s) in the GAM, with the estimated degrees of nonlinearity. The ticks on the x‐axis represent 3000 site combinations. Grey shading depicts the confidence intervals at the 95% level.

For dragonfly species turnover, the deviances explained by the GAM method of the MS‐GDM were relatively low but substantial, 6.52% for ζ_2_ and 27.6% for ζ_5_. The significant predictors for rare species turnover (ζ_2_) included between‐site changes of water temperature, distance, rock cover, shade cover and water dissolved oxygen, which, according to the GLM method of the MS‐GDM, reduced compositional similarity (i.e., increased compositional turnover) as they increased (Figure 4; Data S5 and S6). Of these significant predictors, changes in water temperature and rock cover had linear effects. Changes in dissolved oxygen had a weakly non‐linear effect, and changes in shade cover and distance had highly non‐linear effects on species turnover. For ζ_5_, only changes in water temperature and shade cover had significant, non‐linear effects on species turnover. Increasing changes of both variables reduced dragonfly compositional similarity (i.e., boosted compositional turnover) (Figure 5). In addition, according to the GLM method of the MS‐GDM, changes in rock cover had a linear, negative effect on compositional similarity (Data S5 and S6).

**FIGURE 6:**
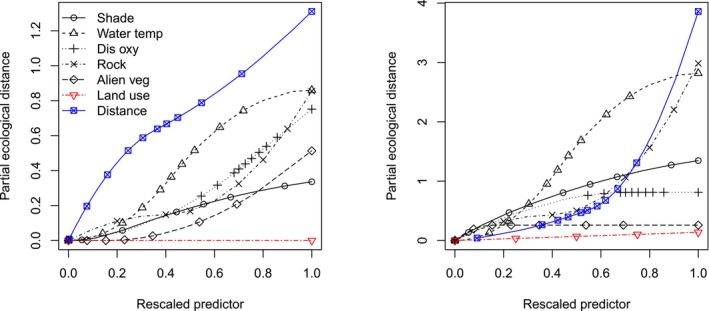
I‐spline regressions of the MS‐GDM for rare species ζ_2_ (left) and common species ζ_5_ (right), with the right‐most magnitude of an I‐spline indicating the contribution of a predictor to compositional turnover. Compositional turnover was measured by normalised zeta diversity (Simpson‐equivalent).

With the I‐spline method of the MS‐GDM, the local response of zeta diversity (Simpson‐equivalent) was fitted across low, medium and high ranges of the predictor variables. The variables explained 7.19% and 10.49% of the deviance in species turnover, driven primarily by rare (ζ_2_) and more common species (ζ_5_), respectively. Changes in land use and invasive alien vegetation cover did not affect dragonfly compositional turnover for either zeta order, which suggests that corridors are functioning analogous to natural habitats. On the basis of the magnitude of the I‐spline response curve, the distance between sites was the most important predictor of turnover for both rare and common dragonflies (Figure 6; Data S7). Dragonfly community turnover for rare species (ζ_2_) was sensitive to variations of distances between nearby sites (low range of distance), medium water temperatures (22.6°C–9.1°C), high rock cover (70%–100%) and medium dissolved oxygen levels (51.6%–75.8%). By contrast, species turnover of more common species (ζ_5_) was sensitive to changes at sites with high rock cover (70%–100%) and medium water temperature ranges (22.6°C–29.1°C) (Figure 6; Data S7). These results can be linked to the landscape features of the various sites and land use types (see Data S8 for site images).

## 4 Discussion

Here, we applied the zeta diversity framework to assess the functioning and value of conservation corridors for conserving dragonfly diversity in a landscape transformed by forestry. Zeta diversity evaluates species turnover across multiple sites (differentiating the effects of rare from those of common species) and is useful for developing management priorities for biodiversity conservation (Hui et al. 2018; Iacarella 2022; Latombe et al. 2017). Our results show that the species richness of dragonflies was slightly but significantly higher in corridors, which could be related to generalist species being able to survive in harsher conditions or that corridors here are being used as both movement and habitat areas. Mean zeta diversity was higher when corridors were included together with natural sites in the landscape compared to having natural sites alone, indicating that corridors can enhance overall dragonfly diversity in transformed landscapes. Our finding that species richness was higher in corridors than in natural sites aligns with that of Rocha‐Ortega et al. (2019), who found that land‐use change can affect odonate community composition without necessarily reducing species richness. This highlights the complex relationship between habitat transformation and biodiversity metrics, where corridors may provide novel habitat combinations that support diverse assemblages while still maintaining essential ecological functions.

Land use (whether a site was in a corridor or a natural grassland) as well as alien plant species cover did not affect turnover of dragonfly assemblages, suggesting that these corridors function similarly to natural grasslands. The presence of the threatened *Chlorolestes apricans* exclusively in corridors is particularly noteworthy. This species is one of South Africa's rarest. It requires sunny, shallow, rocky‐bottom streams with abundant emergent vegetation. Its occurrence in corridors indicates that these managed habitats can provide the specific microhabitat conditions needed by specialised, sensitive species. Similarly, *Allocnemis leucosticta* is a South African endemic and, in our study, was one of the most observed species. It favours either clear, forested streams or open streams with clumps of trees and bushes, conditions that are met in both natural areas and corridors. Establishing such corridors facilitates species movement and creates additional habitats by linking to natural areas, thus promoting regional complexity and habitat heterogeneity, which supports a diverse dragonfly assemblage (Merenlender et al. 2022). As bioindicators and umbrella species, conserving dragonflies will also protect other organisms in the same system, thereby promoting biodiversity (Kietzka et al. 2019; Sahlén and Ekestubbe 2001).

Dragonflies are ectothermic and rely heavily on behavioural thermoregulation to maintain optimal body temperature (Castillo‐Pérez et al. 2022). When controlling for other predictors, species richness decreased with increasing shade cover. The impact of shade on dragonflies is well‐documented. As ectotherms, they rely on external sources for temperature regulation (May 1976, 1991; McGeoch and Samways 1991; Polcyn 1988). Many adult dragonflies tend to avoid shaded areas, indicating that such environments are less favourable for their needs (Kietzka et al. 2021a; Remsburg et al. 2008). Dense shade limits sunlight penetration, reducing suitable habitats for foraging, breeding and thermoregulation (French and McCauley 2018; Remsburg et al. 2008). In our study, shading was likely linked to alien vegetation in both corridors and natural areas, as well as to plantation trees in corridors, all of which replace the open, naturally sunny grasslands. Similar patterns were observed in other South African studies, where the introduction of alien trees decreased species richness, particularly affecting endemic and rare dragonfly species (Samways and Sharratt 2010; Samways and Taylor 2004). Alien trees not only reduce sunlight for thermoregulation but also hinder the establishment of understorey vegetation, limiting prey habitats and perching sites (Renner et al. 2016; Samways and Sharratt 2010).

Dragonfly turnover was primarily driven by stochastic processes, including migration, unpredictable weather events and random dispersal (Assandri 2021). This highlights the importance of considering stochasticity in conservation planning. These random factors could make species incidence highly contingent during a 2 h survey, potentially masking species‐environment relationships that would require long‐term monitoring to quantify. In addition, although the deterministic processes (e.g., competition for sites with high rock cover and medium water temperature) also play a role, there is often a trade‐off between stochastic and deterministic drivers, particularly with more mobile species (de Beer et al. 2023). While our standardised sampling approach ensured comparable detection probability across sites and species estimates were close to the observed number of species, we acknowledge that some occasional, mobile species may have been present but undetected during our surveys.

Although some rare species may not have been detected, our sampling captured a high proportion of the assemblage. We recorded 37 species, closely aligning with a Chao2 estimate of 39, indicating approximately 95% completeness. The mean site‐level richness was 4.03 ± 2.24 species, representing about 10% of gamma diversity. This suggests that within‐site diversity is substantial and representative of the assemblage present during the 2 h surveys. Analyses of compositional turnover require representative rather than exhaustive sampling, and our consistent effort across sites (two observers for 2 h per site) ensured comparability (Chao et al. 2023; Noble et al. 2024). While our sampling design facilitates comparability between our assemblages, it does not ensure that the assemblages are complete or fully representative within sites. We recognise that some rare or transient species may have been overlooked. Nonetheless, the robustness of our results is supported by the comparability of assemblages across sites.

A greater effort may have increased species counts but would not affect the qualitative pattern of turnover. The finding of fewer than one species shared on average between sites is consistent with other odonate studies in heterogeneous landscapes in South Africa (Basel et al. 2024). It reflects specialised habitat requirements rather than solely under‐sampling. Our turnover metrics support this: the order‐2 to order‐1 zeta ratio was 0.20, similar to values reported for African dragonflies. The fitted decline (exp[−1.32 × order]; intercept = 0.93) indicates that most species were site‐restricted, a typical pattern in insect assemblages (McGeoch et al. 2019). We therefore conclude that the dataset is sufficiently representative to provide robust estimates of assemblage turnover, despite the limited number of visits per site.

Geographic distance between sites was the main predictor of compositional turnover for both rare and common dragonflies, suggesting competition or niche differentiation between species. Dragonflies have highly spatially structured assemblages (distance decay of similarity), which are a consequence of their territorial nature, carnivorous diet and strong competitiveness among size classes (Van Buskirk 1992). However, distance may also correlate with unmeasured environmental variables, which is termed the Moran Effect (de Beer et al. 2023). Among rare dragonfly species, half were Zygoptera, which are less mobile, and changes even at short distances between sites significantly enhanced compositional turnover (Daniel et al. 2019; Heiser and Schmitt 2010). The strong influence of geographic distance on compositional turnover aligns with the well‐established concept of distance decay of similarity (Soininen et al. 2007). The similar distance decay patterns observed in both natural areas and corridors suggest that dispersal limitation plays an important role in structuring dragonfly communities across our study landscape, even for these relatively mobile insects, or may indicate that corridors modulate movement in ways comparable to natural landscapes, further supporting their conservation value.

Rare and common dragonfly species were sensitive to changes in water temperatures within the medium range (22.6°C–29.1°C). High turnover may result from species entering or leaving such sites as temperatures change and cross their optimal thermal performance and tolerance ranges (Dallas 2009). We used water temperature and shade as proxies for the microthermal environment, noting that water temperature is strongly correlated with ambient air temperature, e.g., Morrill et al. (2005) found that stream‐water temperature increases by 0.6°C–0.8°C for every 1°C increase in air temperature. There is strong congruency between larval and adult dragonflies, as their territorial nature drives them to select optimal conditions for offspring survival (Kietzka et al. 2021b). Generally, larval growth thrives in warmer water, with optimal temperatures between 21°C and 31°C (Suhling et al. 2015). Warmer temperatures promote higher productivity, leading to rapid egg development and larval growth (Pritchard and Leggott 1987). Compositional turnover of rare species here also responded to changes in medium dissolved oxygen levels. Many rare taxa have narrower physiological tolerances, making them more sensitive to medium dissolved oxygen levels where species sorting occurs. As dissolved oxygen shifts in these ranges, some rare species drop out while others with different tolerances replace them, driving turnover (Mendes et al. 2018; Silva, Azevedo, et al. 2025; Silva, Castro, et al. 2024). Studies in South African rivers similarly show that dissolved oxygen is a key predictor of assemblage composition, with rare and sensitive species responding most strongly (Kietzka et al. 2017). The effect of dissolved oxygen on compositional turnover is known for macroinvertebrates in general (Kefford 1998) and for dragonfly adults and larvae (Kietzka et al. 2017; Mendes et al. 2018) and may also be indicative of the transition between lentic and lotic species (Prescott and Eason 2018).

The turnover of rare and common dragonfly species was significantly influenced by changes in rock cover in highly rocky sites (already with a high range of rock cover). Common species are often strong fliers that can travel long distances to select optimal habitats, permanently or as their requirements change. Rare species are highly habitat‐specific and can be geographically restricted in range, and rocks can act as physical barriers, restricting the dispersal of some species (Korkeamäki and Suhonen 2002; Schindler et al. 2003). Rocks can serve as important foraging perches, basking sites and for thermoregulation (McGeoch and Samways 1991). Dragonflies thermoregulate by selecting suitable microhabitats and using behaviours such as postural adjustment and heliothermy (Castillo‐Pérez et al. 2022; May 1991; McGeoch and Samways 1991). Habitats with rocks positioned in a variety of places (in vs. out of the water, in shade vs. sun) can be important for thermal balance, but can differ between species, which can be linked to differences in body size, colour, dispersal, or thermoregulatory abilities (Ali et al. 2025; Vinagre et al. 2024). Interestingly, differences in shade cover particularly affected turnover of common species, with the strongest effects at intermediate levels. This pattern may reflect the thermoregulatory requirements of widespread dragonfly species, which often benefit from a mosaic of sun and shade that allows for both foraging and temperature regulation. Extremely shaded or open sites may support more specialised communities, while moderate shade levels may host more variable assemblages depending on other habitat characteristics.

## 5 Conclusions

This study demonstrates the significant role of conservation corridors in sustaining dragonfly diversity within transformed landscapes. By enhancing zeta diversity, corridors help counteract the negative impacts of land‐use change on biodiversity and ecosystem integrity (Baselga 2010; Latombe et al. 2017). Through connecting natural areas and increasing regional complexity, they facilitate species movement, gene flow and population resilience (Haddad et al. 2015). Our findings, therefore, highlight the importance of continued investment in the design, management and monitoring of corridors as an effective conservation strategy in a rapidly changing world. Our results showed that dragonfly diversity did not differ between land use types for the sites considered in our study.

The application of the zeta diversity framework proved particularly valuable, revealing subtle but important differences in the compositional structures and drivers of insect biodiversity. This approach offers a powerful tool for refining our understanding of species turnover dynamics and should be applied more widely in biodiversity research (Hui and McGeoch 2014).

It is important to note, however, that our findings apply specifically to corridors incorporating rivers in plantation landscapes of the KwaZulu‐Natal midlands, South Africa. These results should not be uncritically generalised to more degraded or differently managed corridors elsewhere, where conservation effectiveness may differ substantially.

While our study captured key environmental variables known to influence dragonfly communities, we did not directly measure some potentially relevant factors such as corridor width, river depth, or flow velocity. However, these variables have been measured in other studies conducted in the area (see Kietzka et al. 2021a). Corridor width was partially captured through shade measurements (wider corridors typically have less shade). Still, future studies would benefit from explicitly quantifying these additional variables to provide more specific management recommendations.

More broadly, our work speaks to the global insect crisis, which represents a major biodiversity challenge with far‐reaching ecological consequences (Sánchez‐Bayo and Wyckhuys 2019; Wagner et al. 2021). Dragonflies exemplify these declines: they are sensitive indicators of freshwater ecosystem health, yet are experiencing widespread population reductions (Clausnitzer et al. 2009; Ott 2010). By showing that conservation corridors can sustain diverse dragonfly assemblages, including rare and threatened species, this study provides a practical conservation measure that contributes directly to efforts to mitigate the global insect crisis.

## Author Contributions

**Gabriella J. Kietzka:** conceptualization (equal), data curation (equal), formal analysis (equal), project administration (equal), visualization (equal), writing – original draft (equal), writing – review and editing (equal). **James S. Pryke:** conceptualization (equal), funding acquisition (equal), investigation (equal), resources (equal), supervision (equal), validation (equal), writing – review and editing (equal). **Rene Gaigher:** supervision (equal), validation (equal), writing – review and editing (equal). **Michael J. Samways:** conceptualization (equal), funding acquisition (equal), investigation (equal), methodology (equal), resources (equal), supervision (equal), visualization (equal), writing – review and editing (equal). **Cang Hui:** conceptualization (equal), formal analysis (equal), methodology (equal), resources (equal), software (equal), supervision (equal), writing – review and editing (equal).

## Funding

This work was supported by Global Insect Threat‐Response Synthesis (GLiTRS) from the UK Natural Environment Research Council, NE/V007548/1. European Union's Horizon Europe Research and Innovation Programme (B3 ‐ Biodiversity Building Blocks for policy), 101059592. Mondi Ecological Network Programme is supported by Mondi Group, 14493268. National Research Foundation of South Africa, 89967.

## Conflicts of Interest

The authors declare no conflicts of interest.

## Supporting information


**Data S1:** Supporting Information.

## Data Availability

Data are archived at Dryad, and accessible at: https://doi.org/10.5061/dryad.m0cfxppg7.
