# Supplementary material for: Evaluating Conservation Corridor Success for Rare and Common Dragonflies Using Zeta Diversity
Source: Ecol Evol. 2026 Mar 29;16(4):e73251. doi: 10.1002/ece3.73251 (PMC13107279; doi:10.1002/ece3.73251)
Supplement: Supplementary file 1 — Data S1: Supporting Information. [file ECE3-16-e73251-s001.docx]

**Supplementary Information**


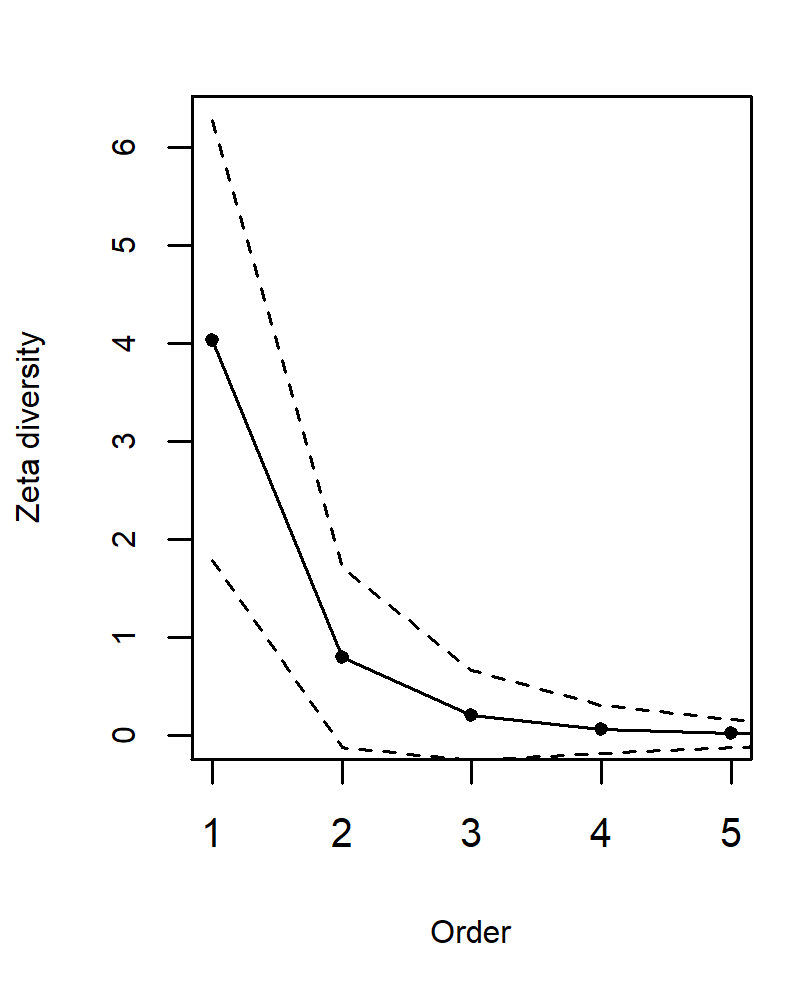


**S1.** The relationship between zeta diversity and the order of zeta shown as zeta diversity decline for natural and corridor sites combined.

**S2.** Species identities and frequencies (from most common to rare) in natural and corridor sites separately and overall.

| Species Name (Authority) | Natural | Corridor | Overall |
| --- | --- | --- | --- |
| *Africallagma Glaucum* (Burmeister, 1839) | 5 | 5 | 10 |
| *Africallagma Sapphirinum* (Pinhey, 1950) | 0 | 1 | 1 |
| *Allocnemis Leucosticta* Sélys, 1863 | 20 | 21 | 41 |
| *Anax Imperator* Leach, 1815 | 5 | 2 | 7 |
| *Anax Speratus* Hagen, 1867 | 7 | 7 | 14 |
| *Ceratogomphus Pictus* Sélys, 1854 | 10 | 6 | 16 |
| *Ceriagrion Glabrum* (Burmeister, 1839) | 3 | 1 | 4 |
| *Chlorolestes Apricans* Wilmot, 1975 | 0 | 1 | 1 |
| *Chlorolestes Fasciatus* (Burmeister, 1839) | 7 | 12 | 19 |
| *Crocothemis Erythraea* (Brullé, 1832) | 0 | 1 | 1 |
| *Crocothemis Sanguinolenta* (Burmeister, 1839) | 1 | 1 | 2 |
| *Elattoneura Glauca* Sélys, 1860 | 12 | 17 | 29 |
| *Ischnura Senegalensis* (Rambur, 1842) | 2 | 3 | 5 |
| *Lestes Plagiatus* (Burmeister, 1839) | 3 | 4 | 7 |
| *Nesciothemis Farinosa* (Förster, 1898) | 2 | 2 | 4 |
| *Notogomphus Praetorius* (Sélys, 1878) | 1 | 7 | 8 |
| *Orthetrum Caffrum* (Burmeister, 1839) | 15 | 7 | 22 |
| *Orthetrum Julia* Kirby, 1900 | 9 | 16 | 25 |
| *Pantala Flavescens* (Fabricius, 1798) | 9 | 1 | 10 |
| *Paragomphus Cognatus* (Rambur, 1842) | 8 | 4 | 12 |
| *Pinheyschna Subpupillata* (Mclachlan, 1896) | 11 | 10 | 21 |
| *Platycypha Caligata* (Sélys, 1853) | 6 | 3 | 9 |
| *Platycypha Fitzsimonsi* (Pinhey, 1950) | 2 | 3 | 5 |
| *Pseudagrion Caffrum* (Burmeister, 1839) | 8 | 18 | 26 |
| *Pseudagrion Citricola* Barnard, 1937 | 1 | 1 | 2 |
| *Pseudagrion Hageni* Karsch, 1893 | 2 | 0 | 2 |
| *Pseudagrion Kersteni* (Gerstäcker, 1869) | 6 | 11 | 17 |
| *Pseudagrion Salisburyense* Ris, 1921 | 8 | 3 | 11 |
| *Pseudagrion Spernatum* Sélys, 1881 | 15 | 23 | 38 |
| *Sympetrum Fonscolombii* (Sélys, 1840) | 3 | 3 | 6 |
| *Tramea Basilaris* (Palisot De Beauvois, 1817) | 0 | 1 | 1 |
| *Trithemis Arteriosa* (Burmeister, 1839) | 1 | 1 | 2 |
| *Trithemis Dorsalis* (Rambur, 1842) | 4 | 3 | 7 |
| *Trithemis Furva* Karsch, 1899 | 19 | 11 | 30 |
| *Trithemis Stictica* (Burmeister, 1839) | 1 | 3 | 4 |
| *Zosteraeschna Minuscula* (Mclachlan, 1896) | 5 | 1 | 6 |
| *Zygonyx Natalensis* (Martin, 1900) | 3 | 2 | 5 |

**S3.** Generalized additive model results showing the contribution of predictors to changes in dragonfly species richness. edf = Effective Degrees of Freedom, Ref.df = Reference Degrees of Freedom, F = F-statistic.

| Variable | edf | Ref.df | F |
| --- | --- | --- | --- |
| Shade cover (%) | 1.17 | 1.31 | 8.83** |
| Water temperature (°C) | 2.40 | 3.04 | 1.85 |
| Dissolved oxygen (%) | 1.00 | 1.00 | 0.79 |
| Rock cover (%) | 1.00 | 1.00 | 0.06 |
| Alien vegetation (%) | 1.00 | 1.00 | 2.53 |
| Land use (t value) | 0.23 | 0.11 | 2.15* |
| Significance levels *<0.05, **<0.01, ***<0.001 | | | |

**S4.** Generalized linear model results showing the contribution of predictors to changes in dragonfly species richness. Est = Estimate of the coefficient, SE = Standard Error of the estimate.

| Variables | Est | SE | t value |
| --- | --- | --- | --- |
| Shade cover (%) | -0.01 | 0.00 | -3.90*** |
| Water temperature (°C) | -0.00 | 0.02 | -0.14 |
| Dissolved oxygen (%) | -0.00 | 0.01 | -0.84 |
| Rock cover (%) | -0.00 | 0.00 | -0.29 |
| Alien vegetation (%) | -0.00 | 0.00 | -1.41 |
| Land use (t value) | 0.24 | 0.11 | 2.20* |
| Significance levels *<0.05, **<0.01, ***<0.001 | | | |

**S5.** Generalized additive model results produced with multi-site generalized dissimilarity modelling (MS-GDM) showing the response of species turnover to changes of predictors, for rare (ζ_2_ = Zeta order 2) and common (ζ_5_  = Zeta order 5) species. edf = Effective Degrees of Freedom, Ref.df = Reference Degrees of Freedom, F = F-statistic.

|  | ζ_2_ | | | ζ_5_ | | |
| --- | --- | --- | --- | --- | --- | --- |
| Variables | **edf** | **Ref.df** | **F** | **edf** | **Ref.df** | **F** |
| Shade cover (%) | 6.66 | 7.77 | 5.97*** | 8.60 | 8.93 | 3.68*** |
| Water temperature (°C) | 1.00 | 1.00 | 15.90*** | 4.77 | 5.85 | 2.46* |
| Dissolved oxygen (%) | 1.43 | 1.74 | 4.44* | 7.32 | 8.30 | 1.78 |
| Rock cover (%) | 1.00 | 1.00 | 12.98*** | 7.46 | 8.10 | 1.81 |
| Alien vegetation (%) | 1.37 | 1.65 | 0.24 | 7.69 | 8.30 | 1.55 |
| Distance (m) | 3.01 | 3.78 | 13.26*** | 7.36 | 7.93 | 1.29 |
| Land use (t value) | -0.06 | 0.07 | -0.85 | 0.01 | 0.60 | 0.02 |

Significance levels *<0.05, **<0.01, ***<0.001

**S6.** Generalized linear model results produced with multi-site generalized dissimilarity modelling (MS-GDM) showing the effect of predictors on species turnover, for rare (ζ_2_ = Zeta order 2) and common (ζ_5_ = Zeta order 5) species. Est = Estimate of the coefficient, SE = Standard Error of the estimate.

| Variables |  | ζ_2_ | | | ζ_5_ | |
| --- | --- | --- | --- | --- | --- | --- |
|  |  | **Est(SE)** | **t value** | **Est(SE)** | | **t value** |

| Shade cover (%) | 0.36(0.11) | 3.13** | 2.35(0.78) | 3.03** |
| --- | --- | --- | --- | --- |
| Water temperature (°C) | 0.70(0.19) | 3.76*** | 2.73(1.20) | 2.29* |
| Dissolved oxygen (%) | 0.59(0.24) | 2.44* | 0.86(1.10) | 0.78 |
| Rock cover (%) | 0.41(0.12) | 3.54*** | 2.34(0.90) | 2.61** |
| Alien vegetation (%) | 0.10(0.15) | 0.67 | 0.00(0.99) | 0.00 |
| Distance (m) | 1.18(0.17) | 6.89*** | 2.14(1.18) | 1.82 |
| Land use (t value) | 0.06(0.07) | 0.89 | 0.18(0.62) | 0.29 |

Significance levels *<0.05, **<0.01, ***<0.001

**S7.** I-spline regression from multi-site generalized dissimilarity models showing the response of Simpson-equivalent zeta diversity to local changes in specific ranges of the predictors, (ζ_2_ = Zeta order 2) and common (ζ_5_ = Zeta order 5) species. Est = Estimate of the coefficient, SE = Standard Error of the estimate.

| Predictors | Range | ζ_2_ | | | ζ_5_ | |
| --- | --- | --- | --- | --- | --- | --- |
|  |  | **Est(SE)** | **t value** | **Est(SE)** | | **t value** |
| Shade cover | Low | 0.00(0.01) | 0.00 | 0.00(0.00) | | 0.00 |
|  | Medium | 0.01(0.02) | 0.52 | 0.01(0.00) | | 3.62*** |
|  | High | 0.07(0.02) | 3.23** | 0.00(0.00) | | 0.00 |
| Water temperature | Low | 0.00(0.02) | 0.00 | 0.00(0.00) | | 0.00 |
|  | Medium | 0.13(0.03) | 4.85*** | 0.02(0.00) | | 6.70*** |
|  | High | 0.00(0.04) | 0.00 | 0.00(0.00) | | 0.00 |
| Dissolved oxygen | Low | 0.00(0.04) | 0.00 | 0.00(0.00) | | 0.00 |
|  | Medium | 0.04(0.04) | 1.04 | 0.00(0.00) | | 0.00 |
|  | High | 0.00(0.03) | 0.00 | 0.00(0.00) | | 0.00 |
| Rock cover | Low | 0.00(0.01) | 0.21 | 0.00(0.00) | | 2.53* |
|  | Medium | 0.00(0.02) | 0.00 | 0.00(0.00) | | 0.00 |
|  | High | 0.10(0.02) | 4.72*** | 0.01(0.00) | | 6.44*** |
| Alien vegetation cover | Low | 0.00(0.01) | 0.00 | 0.00(0.00) | | 0.00 |
|  | Medium | 0.00(0.03) | 0.00 | 0.00(0.00) | | 0.00 |
|  | High | 0.03(0.04) | 0.86 | 0.00(0.00) | | 0.42 |
| Land use | NA | 0.00(0.01) | 0.00 | 0.00(0.00) | | 0.00 |
| Distance | Low | 0.12(0.03) | 4.54*** | 0.04(0.01) | | 7.10*** |
|  | Medium | 0.04(0.03) | 1.24 | 0.01(0.00) | | 3.03** |
|  | High | 0.12(0.05) | 2.28* | 0.00(0.00) | | 0.71 |

Significance levels *<0.05, **<0.01, ***<0.001

**S8.** Selected sites in the KwaZulu-Natal Midlands showing the typical landscape matrices used in our study, comprising plantations, natural areas, and corridors.

**
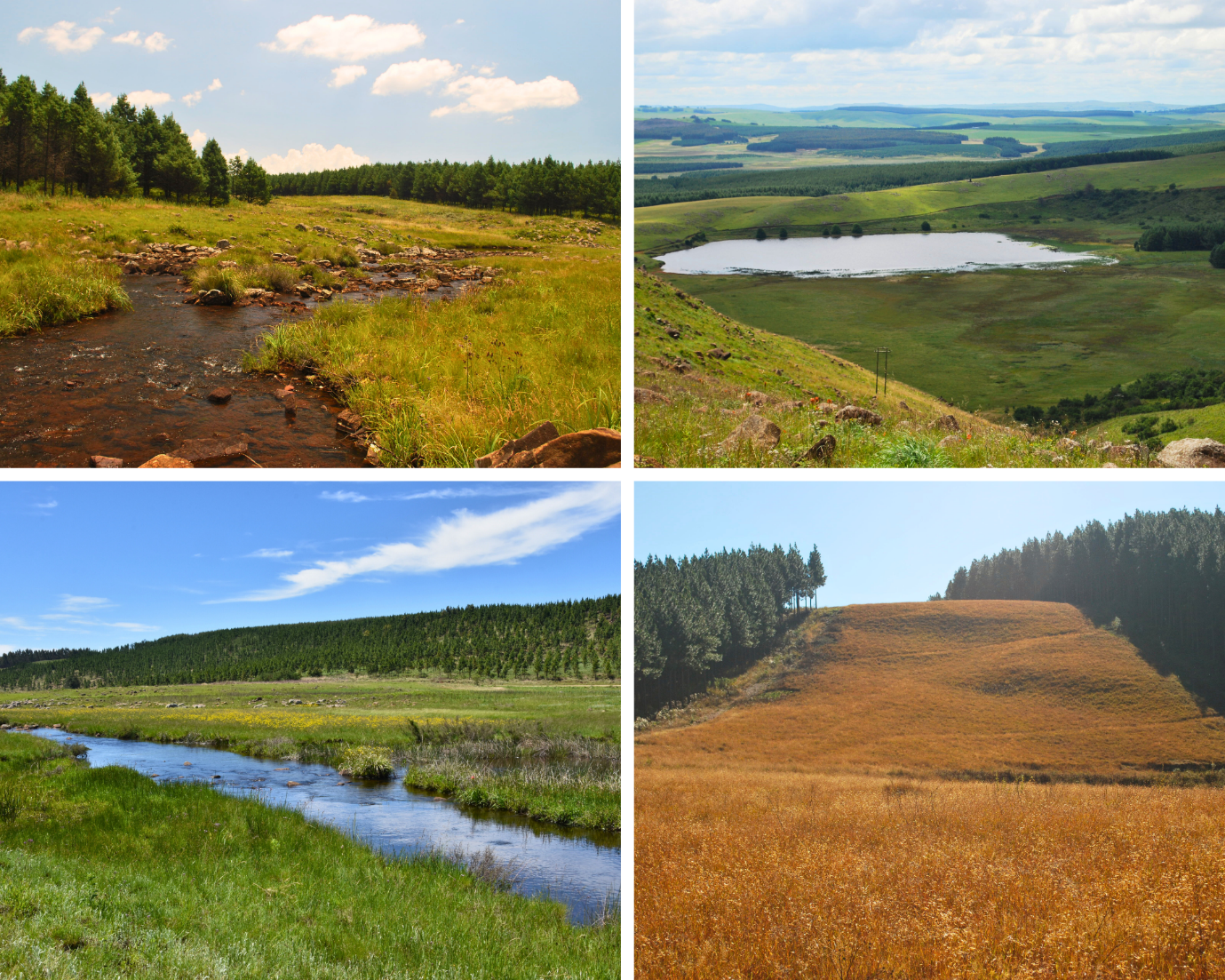
**
